# Adult-Onset Tics After Being Crushed by an Air Conditioner: A Case Report

**DOI:** 10.7759/cureus.45741

**Published:** 2023-09-21

**Authors:** Madeline G Kroeger, Teri Thomsen, Prashant A Natteru

**Affiliations:** 1 Neurology, University of Iowa Hospitals and Clinics, Iowa City, USA; 2 Movement Disorders, University of Iowa Hospitals and Clinics, Iowa City, USA

**Keywords:** tics disorder, tourette's syndrome, tic disorders, post traumatic brain injury, tics

## Abstract

Tics are sudden, repetitive, non-rhythmic movements and/or vocalizations. Generally, tics begin during childhood as a part of Tourette syndrome (TS) and rarely have an onset during adulthood. We describe a 30-year-old male who presented with multiple motor and vocal tics two weeks following a closed head injury with alteration of consciousness as a result of being crushed against the wall by a 4,100-pound air-conditioning unit. He started having motor tics that developed in a rostrocaudal distribution, followed by simple and complex vocal tics. His tics increased in severity over several months following the injury until presentation. He was started on pimozide and received hyperbaric oxygen treatment which improved both motor and vocal tics.

## Introduction

Tics are sudden, repetitive, non-rhythmic muscle movements (motor) and/or vocalizations (phonic or vocal). Tics are often associated with a premonitory sensation (inner pressure/tension/discomfort/itch) that can be suppressed. However, suppression of this sensation increases the urge to complete the tic and relieve the sensation [1]. Tics can be categorized as simple or complex. Simple tics (either motor or vocal) are characterized by a brief duration and are seemingly out of place or unnecessary. Simple motor tics can include things like quick movements of the eyes, limbs, or head. Simple vocal tics can include additional sounds such as hums, grunts, gasps, or clearing of the throat. Complex tics are characterized by multiple consecutive motions or sounds. Complex motor tics include a series of facial and head movements, tapping, or jumping. Complex vocal tics can include full words or phrases as well as palilalia, echolalia, or coprolalia. Typically motor tics precede vocal tics in patients who develop both [2].

Generally, tics begin during childhood as part of Tourette syndrome (TS) which onsets in a rostro-caudal and proximo-distal manner. TS is more common in males and is sometimes associated with attention-deficit/hyperactivity disorder (ADHD) and obsessive-compulsive disorder (OCD) [2]. Tics rarely onset during adulthood [2]. Adult-onset tics can be roughly categorized into three groups: primary or idiopathic tics, secondary tics or “tourettism”, and childhood-recurrent tics. Secondary tics can be caused by a multitude of mechanisms including brain injury, drug use, and some neurodegenerative diseases [3]. Tics developing following a traumatic brain injury (TBI) will be the focus of this case report.

This article was previously presented as a meeting abstract at the XXVIII World Congress on Parkinson’s Disease and Related Disorders on May 15, 2023.

## Case presentation

A 30-year-old man was crushed against the wall by a 4,100-pound air-conditioning unit at his work, sustaining a mild TBI with alteration of consciousness. Two weeks after the injury, he developed motor tics in a rostrocaudal and proximo-distal pattern, followed by simple and complex vocal tics. He presented to our clinic about 10 months after the onset of tics. His motor tics were characterized by brief twitches of his neck that would extend to his left shoulder and arm. His motor tics are mostly discrete but occasionally can be sequential with the involvement of his legs. His vocal tics can be simple (making an “ah” sound; clearing his throat) or complex - palilalia (repeating words like “cheese”, and “I’m sorry”). At times, he would use profanities as a part of coprolalia as well. The patient endorsed a sense of relief after the completion of his tics. He had no personal or family history of tics, ADHD, or OCD. At the time of his injury, computed tomography (CT) of the head showed no evidence of intracranial pathology while CT cervical spine showed bilateral acute non-displaced fractures through the posterior ring of the C1 vertebra, along with chip fracture of the left spinous process at C6 vertebra.

His tics had increased in severity over several months to a frequency of 10-15 tics per minute, interfering with his functional ability. At presentation, his neurological examination was unremarkable except for the motor and vocal tics. His total tic severity score was 38 and the Total Yale Global Tic Severity Scale (YGTSS) was 88. Due to concerns about blood pressure variability, alpha agonists like clonidine and guanfacine were avoided. Instead, he was treated with a dopamine receptor blocker (pimozide 2mg twice daily). At the two-month follow-up appointment, there was a significant improvement in both his motor and vocal tics, as evidenced by the reduction in the total tic severity score to 18 and YTGSS to 38 (Video 1). For his TBI, he underwent two sessions of hyperbaric oxygen therapy (HBOT) at pressures of 2 atmospheres absolute (ATA).

**Video 1:** Tics improved with pimozide

## Discussion

Tic disorders can be classified as primary and secondary tic disorders. Under primary, three subcategories exist based on duration and age of onset: provisional, persistent/chronic, and Tourette’s. Provisional tic disorder encompasses single or multiple motor and/or vocal tics present for less than a year, while persistent/chronic tic disorder involves either motor or vocal tics for more than a year duration. TS requires both motor and vocal tics for at least a year. All these require an onset prior to 18 years of age [4].

Tic disorders rarely onset in adulthood, but a few cases have been described of their emergence following TBI. These cases may represent an unmasking of underlying mild tic disorder originating in childhood (recurrence) or a result of changes to brain circuitry following the injury (secondary tic disorder). Dysfunctions in the cortico-basal ganglia-thalamo-cortical circuitry have often been attributed to the pathogenesis of tics in TS and it is theorized that secondary causes of tics also affect this circuitry [5]. Therefore, with post-traumatic tics, damage to the circuitry may be direct or could be more nuanced through the delayed effects of hemosiderin deposits or abnormality in the neuronal plasticity related to the healing process following trauma [1,6,7]. 

There is literature suggesting that there may be two categories of post-traumatic tics based on the proximity of onset to the injury. De Souza et al. suspect that patients with mild TBI who have tics manifesting within five months of injury may have slight underlying features or a family history of tics, ADHD, or OCD [1]. These patients typically respond well to current treatments. The other category is tics that have a delayed onset after severe TBI with potentially abnormal head imaging. The underlying pathology of this category of patients is thought to be a delayed effect of TBI on the aforementioned circuitry, or aberrant neuro-regeneration [8]. These patients, unfortunately, are less likely to respond to treatment [1]. We have summarized the cases published so far with post-traumatic tics in Table 1.

**Table 1:** Cases of post-traumatic tics MVA = motor vehicle accident; LoC = loss of consciousness

Author	Age at Injury / Gender	Injury	Time to tic onset	Tic type	Treatment	Outcome
Eriksson et al. [9]	3 y.o. M	MVA, R temporal fracture	"Shortly thereafter"	Tics affecting eyes, twitches of face/shoulders, cheek/tongue bites, vocal tics.	Phenytoin, Diazepam, Barbiturates	Resolution
Majumdar et al. [10]	7 y.o. F	MVA with LoC for 1 week	15 months	Blinking, shoulder/arm jerks, trunk flexing, with vocal tics including grunting, tongue clicks, coprolalia, echolalia, and echopraxia with partial suppression.	Haloperidol	Improvement
Fahn [11]	18 y.o. M	Head strike by steel girder w/LoC for a few minutes	"Couple of months"	Twitches of face, eyes, neck, limbs, and vocal tics like grunts, sniffs, throat clearing, snorting, and whistling. Partially suppressible with rebound exacerbation.	Haloperidol, Clonazepam	Improvement
Ranjan et al. [12]	19 y.o. M	MVA with LoC for 1 week	1 year	Face grimacing, sniffing, eyelid twitches with vocal tics including grunting and throat clearing with transient suppression.	None	Spontaneous improvement over 1 year
Kumar et al. [13]	21 y.o. M	MVA with LoC for a few minutes	5 months	Neck jerks, shoulder shrugging, back arching, and eye twitching that is suppressible but with post-suppression exacerbation.	Clonazepam, clonidine	Minimal improvement
Krauss et al. [7]	21 y.o. M	MVA with LoC for 3 weeks	“Within months” after 2nd MVA	Nose picking, rubbing of nose/eyes, sniffing, leg twitches.	Fluoxetine	Improved mood
Krauss et al. [7]	23 y.o. M	MVA with no LoC	1 day	Jerking of head to the right, twitches of the platysma, lip smacking, and facial grimacing that is transiently suppressible with post-suppression exacerbation.	baclofen, cyclobenzaprine, carbamazepine, haloperidol, buspirone, trihexyphenidyl, clonazepam, tetrabenazine, botulinum toxin.	Improvement with tetrabenazine and botulinum toxin only.
Singer et al. [14]	25 y.o. M	MVA, cerebral concussion w/LoC for 45 minutes	2-3 weeks	Repetitive movements of periocular muscles, face, head, neck, right shoulder.	Lorazepam, haloperidol, clonidine	No improvement
De Souza et al. [1]	28 y.o. M	Nail penetration in the skull	7 years	Head turning and vocal tics including involuntary vocalizations and coprolalia.	Valproate, risperidone	Improvement with risperidone
Siemers et al. [15]	33 y.o. M	3 story fall w/o LoC	2 weeks	Small amplitude head and neck movements with transient suppression.	Haloperidol, pimozide	Improvement
Gaul [16]	35 y.o. M	MVA, head injury w/o LoC	2 weeks	Large amplitude twitching of platysma with partial suppression.	Clonidine, diazepam	Improvement
Krauss et al. [7]	47 y.o. M	MVA with LoC briefly	4 days	Left face and neck twitching, eye blinks, head bobbing with vocal tics including grunting/sniffing; transient suppression	Oxycodone, codeine, acetaminophen, diazepam, flurazepam, promethazine, meperidine, clonazepam	No improvement
Poulopoulos et al. [17]	Unknown, M	MVA with LoC for 1 month	2 months	Repetitive right shoulder flexion, elbow/wrist extension, truncal contractions, left torticollis, blepharospasm, sniffing, and vocal tics including throat clearing.	Haloperidol, pimozide, trazodone, clonidine, sertraline, levodopa, baclofen, tetrabenazine	Improvement with tetrabenazine

Our case may fall under the first category since tics emerged shortly after injury with normal neuroimaging and good treatment response, but there was no reported history of tics, ADHD, or OCD. A review of the case reports of post-traumatic tics revealed that most patients do not have a personal or family history of tics or related conditions. Additionally, 37.5% of the cases reviewed had negative head imaging [2]. Another study suggests that even patients with intracranial findings on brain imaging do not have direct basal ganglia damage [12].

Patients with tics often experience an altered quality of life and psychosocial sequelae. About 90% have at least one comorbid condition in their lifetime with OCD and ADHD being more common [4]. Just like TS, patients with tourettism experience similar comorbidities. One study suggests that 52.2% of patients with secondary tic disorders experience features of OCD and 34.8% experience features of ADHD [18].

With regards to the management of TS and tourettism, a wide variety of modalities exist. Historically, medical options including alpha-2 agonists (clonidine, guanfacine), dopamine receptor blockers (risperidone, aripiprazole, haloperidol, pimozide, fluphenazine) as well as others (clonazepam, tetrabenazine) have been used [19]. Of late, comprehensive behavioral intervention for tics (CBIT) has emerged as a first-line therapeutic option for tic disorders [4]. Procedural treatments include botulinum toxin injections for focal motor or phonic tics as well as deep brain stimulation for cases refractory to medical management [19]. For TBI, HBOT can be considered as it has proved to inhibit apoptosis, promote angiogenesis and neurogenesis, and suppress inflammation eventually causing neuroprotection [20]. Given the wide range of options, it is important to consider specific patient goals in designing a treatment plan. Our patient was offered both options of CBIT and medical therapy, and the latter was chosen.

## Conclusions

Tic disorders rarely start in adulthood, but a few cases have been described of their emergence following TBI (secondary tics or “tourettism”). The emergence of tics post-TBI may represent an unmasking of an underlying mild tic disorder or are a result of alterations to the cortical-subcortical circuitry following the injury causing a release phenomenon. Clinically, they tend to mirror the rostral-caudal, proximo-distal, and motor-to-vocal patterns as seen in TS. The prognosis of post-traumatic tics depends on the proximity of tics from the injury, with early onset (less than five months from the injury) tend to respond well to medical and behavioral therapy. 
